# High infection risk of intestinal helminths despite WASH interventions: A cross-sectional study in Khammouane province, Lao PDR

**DOI:** 10.1371/journal.pntd.0014388

**Published:** 2026-06-01

**Authors:** Vilaysone Khounvisith, Siriphone Virachith, Nouna Innoula, Bounta Vongphachanh, Latdavone Khenkha, Jan Hattendorf, Somphou Sayasone, Judith M. Hübschen, Antony P. Black, Peter Odermatt

**Affiliations:** 1 LaoLuxLab/Vaccine Preventable Diseases Laboratory, Institut Pasteur du Laos, ‌‌Vientiane, Laos; 2 Swiss Tropical and Public Health Institute, Allschwil, Switzerland; 3 University of Basel, Basel, Switzerland; 4 Lao Tropical and Public Health Institute, Vientiane, Laos; 5 Department of Infection and Immunity, Luxembourg Institute of Health, Esch-sur-Alzette, ‌‌Grand-Duchy of Luxembourg; 6 School of life sciences, University of Westminster, London, United Kingdom; Xinjiang Medical University Affiliated First Hospital, CHINA

## Abstract

**Background:**

Despite the widespread implementation of water, sanitation and hygiene (WASH) program over the last two decades, Lao People’s Democratic Republic (Lao PDR) remains highly endemic for intestinal helminth infections. We assessed the intestinal helminth infections burden and the link with WASH levels in an area with WASH interventions.

**Method:**

A cross-sectional study was conducted across 81 villages in Khammouane province, Lao PDR, where WASH interventions were implemented. Villages and 30 participants (aged 18–87 years) per village were randomly selected. Demographics, individual and household data, including WASH-related factors were collected. Stool samples were examined for intestinal helminth infections using formalin-ethyl acetate concentration technique (FECT). Logistic regression analysis was performed to identify risks associated with intestinal helminth infections.

**Results:**

A total of 1530 participants were included. Multi-helminth species infection was very common, e.g., 30.1% had a double infection. *Opisthorchis viverrini* was most prevalent (56.9%), followed by hookworm (54.7%), minute intestinal flukes (22.7%), and *Trichostrongylus* spp. (10.7%). Overall, 56.1% and 44.4% had access to safely management water services and sanitation facilities, respectively, and 17.7% practiced open defecation. Improved WASH levels were not associated with reduced intestinal helminth infections. District of residence, age, gender and raw food consumption predicted helminth infections. E.g., *O. viverrini* infection was associated with residency in Mahaxai district (Mahaxai, aOR = 14.0; 95% CI = 9.5-20.6; p < 0.001 and Bulapha, aOR = 3.0; 95% CI = 2.0-4.4; p < 0.001).

**Conclusion:**

Our findings indicate a high intestinal helminth infections burden in this province, with several risk factors such as location, age, gender and consumption of raw food predicting helminth infection. Improved WASH levels are not associated with reduces prevalence of intestinal helminth infection in this cross-sectional study.

## Author summary

Intestinal helminth infections remain a major public health concern in Lao PDR despite long-term investments in water, sanitation, and hygiene (WASH) programs. In this study, we assessed the burden of intestinal helminth infections and their association with WASH conditions in Khammouane province, where WASH interventions have been implemented. We conducted a cross-sectional survey across 81 villages and analysed stool samples from 1530 adults. We found a high prevalence of infections, with Opisthorchis viverrini and hookworm being the most common and co-infections frequently observed. Although more than half of the participants had access to improved water and sanitation services, these factors were not associated with a reduced risk of infection. Instead, demographic and behavioural factors, particularly geographic location and consumption of raw or undercooked food were key findings. Our results suggest that WASH interventions alone are insufficient to control intestinal helminth infections in this setting. Integrated strategies that include health education and behavioural change are essential to effectively reduce transmission.

## Introduction

Intestinal helminth infections are a significant global health concern, particularly in areas with poor sanitation, inadequate access to clean water, and limited healthcare resources [1]. Intestinal helminth infections may lead to a wide range of health issues, from mild gastrointestinal disturbances to severe malnutrition, anaemia, impaired cognitive development, and liver cancer (cholangiocarcinoma) [2].

Indeed, the link between Water, Sanitation, and Hygiene (WASH) and intestinal helminth infections is well established. Reports indicate that improving socio-economic conditions, including access to clean water, proper sanitation, and better hygiene practices, can significantly reduce the prevalence of intestinal helminth infections [3,4]. In Southeast Asia, the most common intestinal helminth infections are *Ascaris lumbricoides* at 18%, followed by *Trichuris* at 14% and hookworm at 12% [5]. *Opisthorchis viverrini (O. viverrini)* is also widely prevalent. E.g., in high-risk villages in central and southern Lao People’s Democratic Republic (Lao PDR), more than half of the population is infected with *O. viverrini* [6,7]*.*

In Lao PDR, the population faces challenges in WASH, particularly in rural areas where access to clean water and sanitation facilities is limited [8]. In Lao PDR, in 2023, 4.20% had no access to safe improved drinking water sources, almost 12.8% had no access to improved sanitation facilities, and almost 4.7% had no access to handwashing facilities [9]. Intestinal helminth infections are highly endemic in the country. Since 2005, the national helminth control program has been implementing routine deworming for primary school children across the country [10]. In 2019, a national survey revealed that the prevalence of intestinal helminth infections among adults is substantial, with 21.6% infected with hookworm, 18.8% with *O. viverrini* and 4.8% with *Strongyloides stercoralis* [11].

Despite the continued presence of intestinal helminth infections, there has been a noticeable decline in prevalence over the past decade [6,11]. Hence, the reduction of intestinal helminth infections might be attributed to some extent to the implementation of WASH interventions across the country. However, the effectiveness of such interventions can vary considerably depending on the local context. WASH interventions aim to provide access to clean water and adequate sanitation, as well as to raise awareness of the crucial role of hygiene. While the overall decrease in intestinal parasitic infections is encouraging, the contribution of WASH intervention remains unclear and is rarely assessed.

In areas with suboptimal WASH infrastructure, the risk of reinfection and continued transmission may remain high, undermining the gains achieved through deworming programs. Therefore, in an area where WASH interventions had been conducted, we aimed (i) to assess the intestinal helminth infection burden and (ii) to explore the link between intestinal helminth infection and WASH levels. To that end, we conducted a cross-sectional study in three districts of Khammouane province in central Lao PDR.

## Methodology

### Ethics statement

The study complied with the requirements of the Helsinki declaration on health research. The study was approved by the National Ethics Committee for Health Research (NECHR), Ministry of Health (MoH), Vientiane, Lao PDR (NECHR Ref. No. 041/2022). Permission to conduct fieldwork was obtained from the Ministry of Health, as well as from the Provincial Health Office of Khammouane Province and the District Health Offices in Nakaiy, Bualapha, and Mahaxai districts. Study participants were informed about the study procedures as well as the benefits and risks, and their voluntary participation. Written informed consent was obtained from each study participant. Participants diagnosed with parasitic infections received anti-parasitic medication from each district hospital according to the Lao national treatment guidelines [12].

### Study area

Our study was conducted in Khammouane Province, located in central Lao PDR, bordering Bolikhamxay and Savannakhet provinces to the North and South, respectively. The province is divided into 10 districts and comprises 569 villages, with an estimated population of approximately 434,000 residents [13]. Khammouane province has a high ethnic diversity, including significant populations of Lao, Phou Thay, and Brou communities [14]. These groups exhibit distinct cultural practices and behaviours related to WASH [14]. In 2016, a WASH intervention was performed by the Lao-Luxembourg Development Cooperation in 34 villages across three districts: Nakaiy, Mahaxai and Bualapha. It comprised supporting the construction of toilets in households, providing hygiene education and improving access to safe drinking water. Moreover, they also gave support to update water supplies, set up water management communities for long-term usage within the villages and for maintenance of household latrines [15]. We selected the three WASH intervention districts – Nakai, Mahaxai, and Bualapha – as our study area.

### Study design and population

**Fig 1:**
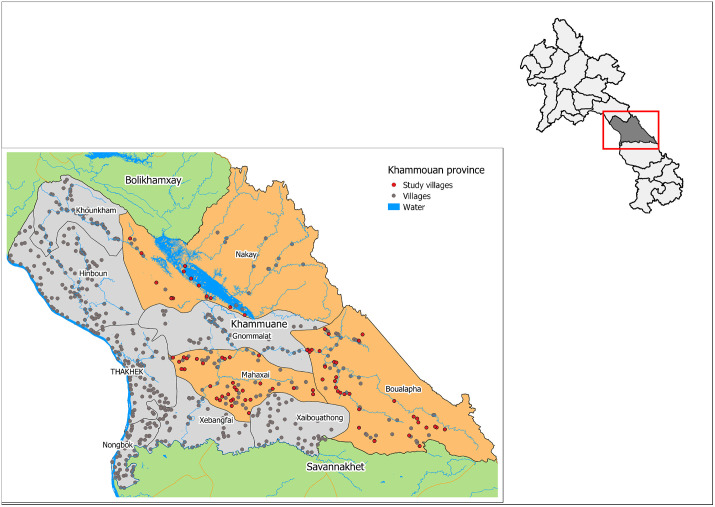
Map of Khammouane province showing the study area and study villages sampled in Nakaiy, Mahaxai and Bualapha districts (orange). The map was created using QGIS Geographic Information System (Open Source Geospatial Foundation Project. Version 3.4: https://qgis.org). The Lao administrative boundary shapefile was obtained from the geoBoundaries open license CC-BY 4.0 (https://www.geoboundaries.org/countryDownloads.html).

**Fig 2:**
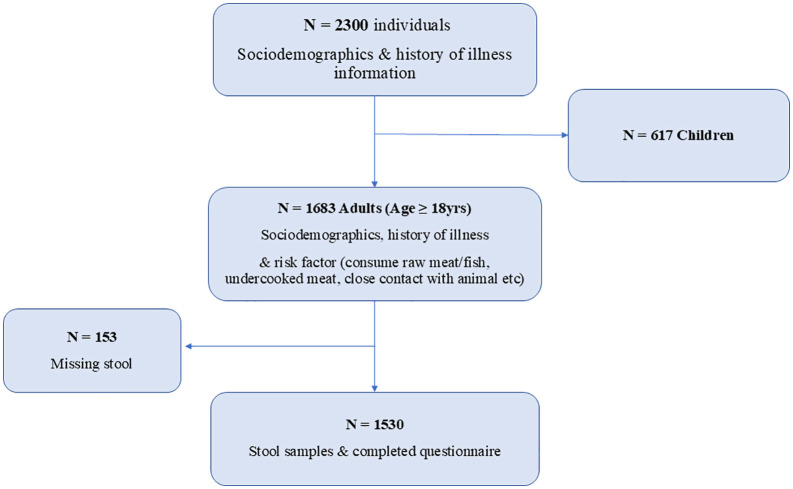
Flowchart of participant recruitment.

Our cross-sectional study was part of a larger study assessing the association of viral infections with WASH exposures that has been described elsewhere [16]. In brief, cluster sampling was used to select households within the three districts: 14 villages in Nakai, 34 villages in Mahaxai and 34 villages in Bualapha, where previously WASH interventions took place in Khammouane Province. Villages were selected using simple random sampling from all villages in accessible areas, selecting 81 out of 174 villages. Within each selected village, households were randomly chosen from the household list, and all household members were recruited from these households until 30 participants were recruited from the village (Fig 1). A total of 2430 individuals were assessed for eligibility, 130 declined to participate and 2300 were enrolled in the larger study. Out of these individuals, 1530 adults from 80 villages met the inclusion criteria for assessing parasite infestation as described in this manuscript (Fig 2).

All individuals aged 18 years and older from the selected households were eligible. Interviews were conducted by investigators at the village head’s office, temples or another convenient village location. The inclusion criteria were (i) age 18 years and older who provided consent, (ii) voluntarily agreed to participate (iii) able to provide one stool sample. Exclusion criteria were (i) pregnancy, (ii) presence of acute illnesses such as fever, acute gastroenteritis (diarrhea or vomiting), common cold, pneumonia, etc, (iii) patients under chemotherapy, (iv) individuals unable to provide a stool sample, (v) nonconsenting individuals (vi) children aged less than 18 years old.

The questionnaire administered to the participants and was structured into four sections: a) sociodemographic data, which included details such as age, sex, ethnicity, education, occupation and family income per month and social-economic status (SES), b) knowledge, attitude and practice toward WASH, c) history of illness, including infectious diseases and d) risk factors related to intestinal helminth infection, e.g., consuming raw food, raw fish, etc.

### Field and laboratory procedures

Before initiating sample collection, an official request letter was provided to the provincial and district hospitals. The district hospital then communicated with the village head, who subsequently informed the community members. During the village visit, the stool containers were distributed to the heads of families, each labelled with the name of the corresponding family member. Instructions on stool collection were provided. The containers were collected the following day, and the questionnaires were administered.

At the district hospital, from each sample, approximately 2g of stool was fixed in 10 ml of sodium acetate acetic-acid formalin. The fixed samples were stored at room temperature and transported to the Lao Tropical and Public Health Institute in Vientiane capital. The formalin-ethyl acetate concentration technique (FECT) was used to detect the presence of intestinal helminth and protozoa infection [17]. All intestinal helminth eggs observed under the light microscope were identified, counted and recorded separately by species in a blinded manner by two investigators. Approximately 10% of the results were discrepant and were discussed among the laboratory experts and lab technicians to reach a consensus.

### Statistical analysis

Data were collected in the field using tablets with the Kobo toolbox [18]. The analysis was performed using STATA software, version 16.0. Descriptive analysis was conducted on all variables, including district, age group, sex, ethnicity, education, occupation, WASH-levels and the risk factors, and were described using the number of observations (n), mean or median, interquartile range (IQR), and standard deviation (SD).

Data on WASH levels were collected through interviews using a structured questionnaire administered to the head of each household. We did not validate the WASH levels by investigator observations. The resulting scores were subsequently categorized into levels based on the UNICEF/WHO guidelines [19] (Table 1).

**Table 1:** WASH levels scores base on UNICEF/WHO [19].

Variables	WASH levels scores				
**1**	**2**	**3**	**4**	**5**
Water source service level	Surface water	Unimproved	Improved: Limited	Improved: Basic	Improved: Safely managed
Sanitation service levels	Open defecation	Unimproved	Improved: Limited	Improved: Basic	Improved: Safely managed
Hygiene service levels	No facilities	Limited	Basic	N/A	N/A

Legend: Water source service levels: 1). Surface water: drinking water directly from a river, dam, lake, pond, stream, canal or irrigation canal; 2). Unimproved: drinking water from an unprotected dug well or unprotected spring; 3). Improved limited: drinking water from an improved source that requires a journey of more than 30 min round trip, including queuing; 4). Improved basic: < 30 min including queuing; 5). Improved safely managed: water source located on the premises, available when needed, free from faecal and priority chemical contamination. Sanitation service levels: 1). Open defecation; 2). Unimproved: Use of pit latrines without a slab or platform, hanging latrines or bucket latrines; 3). Improved limited: use of improved facilities shared between two or more households; 4). Improved basic: not shared with other households; 5). Improved safely managed: not shared with other households and excreta are safely disposed of *in situ* or transported and treated offsite. Hygiene service levels: 1). No facility: No handwashing facility on premises; 2). Limited: Availability of handwashing facility lacking soap and/or water at home; 3). Basic: Availability of a handwashing facility on premises with soap and water. For statistical analysis of water and sanitation levels, the classification of “improved” levels grouped “limited”, “basic” and “safely managed” together.

The SES of the population was assessed using a household-based asset approach [20]. The participants were grouped into three tertiles: (i) poor, (ii) middle, and (iii) high SES using a principal composite assessment (PCA) analysis [20]. The PCA was calculated on a range of household assets, housing characteristics (materials used for the roof, floor, and walls), access to electricity, and ownership of various items, including televisions, radios, refrigerators, cars, bicycles, and motorcycles. Moreover, land use and livestock ownership (buffalo, cows, pigs, goats, and poultry) were included in the analysis to provide a comprehensive assessment of household wealth and SES.

Bivariate and multivariate mixed effect logistic regression models were used to examine associations between helminth infection status and socio-demographic factors, WASH levels, and other risk factors. The village was included as a random effect to account for potential correlation within villages.. In the multiple logistic regression analysis, intestinal helminth infection was the outcome variable and reported risk factors were independent variables. Variables with a p-value of less than 0.2 in the bivariate analysis were included in the multi-variable model, along with additional key risk factors that were deemed clinically important, such as: having domestic animals near the house, using animal stool as fertilizer, cleaning nails, and consuming vegetables, regardless of their statistical significance in the univariate analysis (S2 Table). Multicollinearity among independent variables was assessed using variance inflation factors (VIF) and variables with VIF > 10 were considered to have significant collinearity. The analysis reported crude (cOR) and adjusted (aOR) odds ratios and 95% confidence intervals (CIs) for each variable. A p-value of less than 0.05 was considered statistically significant.

## Results

### Socio-demographic characteristics

A total of 1530 adult study participants had a complete dataset and stool samples (Fig 2). They originated from three study districts of Khammouane province: Nakaiy 15.0%, Mahaxai 50.8% and Bualapha 34.2%. More than half of the participants were female 55.2% (average age 39.6 ± 12.7 years, range 18–87 years). Almost 60% of participants (57.7%) identified as Lao-Loum. About one third (32.0%) of participants had no formal education, 39.5% completed primary school, and 2.4% achieved a university degree. Farming was the primary occupation for 91.1% (Table 2).

**Table 2:** Socio-demographics characteristics of study participants, n = 1530.

Variables	Total
	n=1530 (%)
District	
Nakaiy	230 (15.0)
Mahaxai	777 (50.8)
Bualapha	523 (34.2)
Sex	
Male	685 (44.8)
Female	845 (55.2)
Age (years), mean (SD)	39.6 (±12.7)
Age group	
18-20	65 (4.3)
21-30	336 (22.0)
31-40	481 (31.4)
41-50	339 (22.2)
>50	309 (20.2)
Ethnicity	
Lao-Tai (Lao-Loum)	883 (57.7)
Mong-Khmer (Lao Theung)	647 (42.3)
Levels of education	
No schooling age	489 (32.0)
Primary school	605 (39.5)
Upper 2nd school	268 (17.5)
Lower 2nd school	131 (8.6)
University/bachelor	37 (2.4)
Occupation	
Student	9 (0.6)
Farmers/Housewife	1394 (91.1)
Office staff/commerce/business	127 (8.3)
Wealth Index measurement (n = 1513)	
Poor tertile	450 (29.8)
Middle tertile	532 (35.2)
High tertile	531 (35.1)

### Water and sanitation status of the households

WASH levels in this study district have been previously reported [16]. In brief, overall, 56.1% of the population used safely managed water services, 24.1% relied on limited water services and 9.9% depended on surface water sources. For sanitation, 44.4% had access to safely managed facilities, 26.6% to basic facilities, while 17.7% still practiced open defecation. Regarding hygiene, 69.2% of the population practiced basic ~ handwashing with soap, whereas about 7.7% had no hygiene facilities at home (S1 Table).

S2 Table provides detailed results on additional risk factors. In summary, 26.6% of the study population reported insufficient overall water availability and 22.3% specifically reported inadequate drinking water. Annual flooding affected 28.3% of households, with rivers identified as the primary source in 79.9% of cases. Dietary risk factors were prominent: 54.5% consumed raw meat, 60.7% consumed undercooked meat, and 73.3% consumed raw fish. Consumption of raw shellfish (5.6%) and snails (13.3%) was less common. Nearly all participants (97.4%) reported consuming raw vegetables. Barefoot activities were rarely reported (1.3%), whereas fingernail trimming was common (81.4%). Almost half of participants had livestock near their homes (48.2%), and 47.3% reported pig rearing.

### Intestinal helminth infections

**Fig 3:**
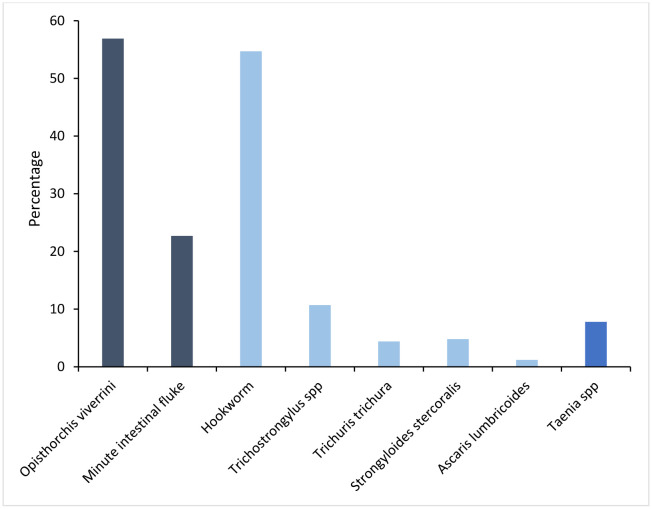
The prevalence of intestinal helminth infection. Dark grey represented trematodes, light grey represented nematodes and light blue represented cestodes.

*O. viverrini*, MIF, hookworm, and *Trichostrongylus* spp. were the most frequently diagnosed helminth species. *O. viverrini* was the most prevalent infection with 56.9% of the participants infected (Fig 3).

Hookworm was diagnosed in 54.7% of the participants. *Trichostrongylus* spp*.* was found in 10.7%. All of these had 1–18 eggs, and were classified as “light intensity”. *T. trichiura* was present in 4.4%; 67/1530. *Strongyloides stercoralis* was detected in 4.8%; 73/1530. Other nematode infections showed lower prevalences such as *Ascaris lumbricoides* (1.2%; 18/1530) and *Enterobius vermicularis* (0.8%; 12/1530).

*Taenia* spp*.* was detected in 7.8%; 119/1530 of the participants. The complete list is shown in the S3 Table. No noteworthy differences among age categories were found for any helminth infection (S3 Table).

**Fig 4:**
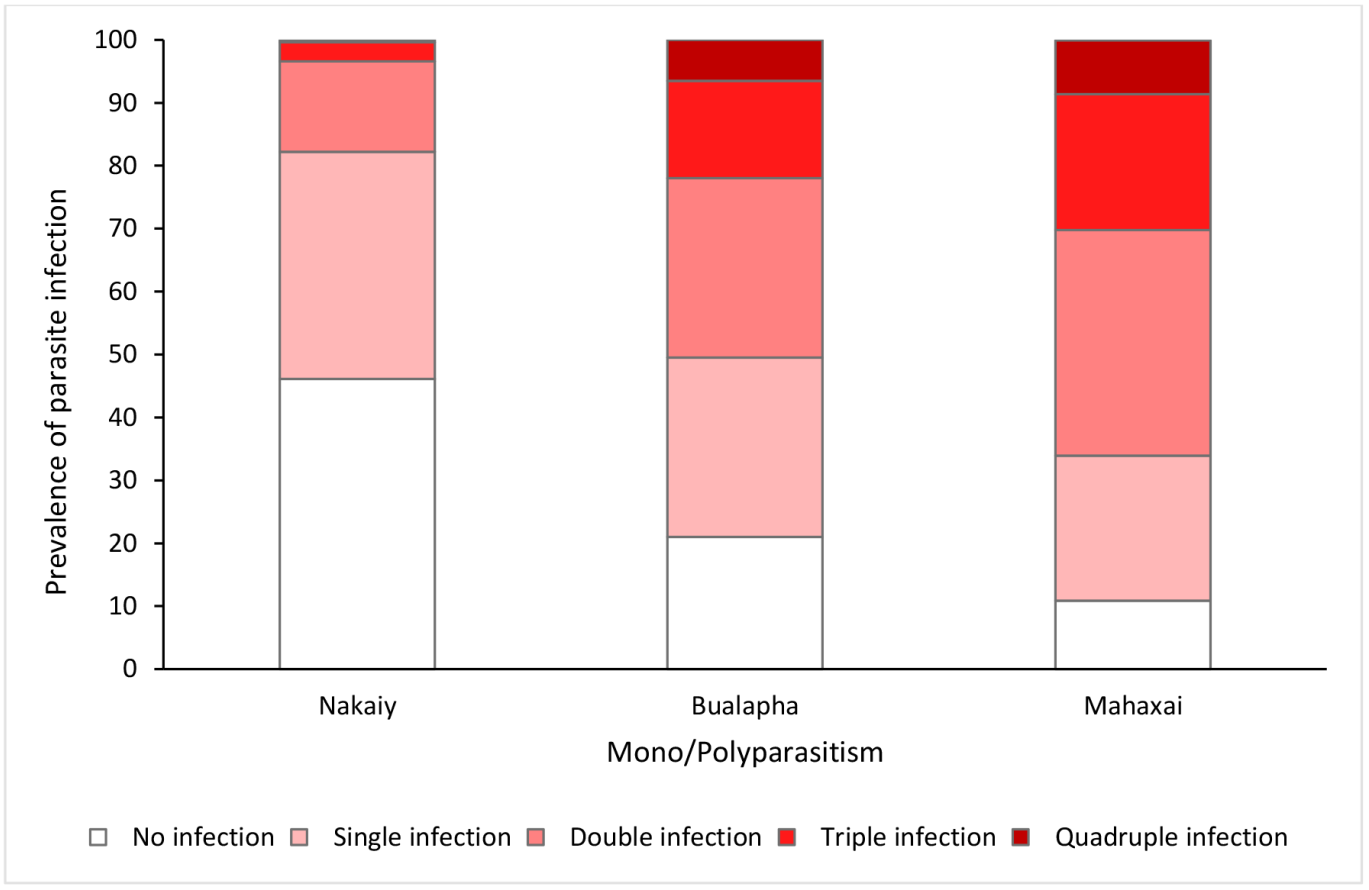
The mono/polyparasitic infection by district, n = 1530.

Infection with multiple helminth species was very frequent. Double, triple, and quadruple infection occurred in 30.1%, 16.7%, and 6.6% of participants, respectively, while 26.9% had a single helminth species infection, and 19.7% were of any infection (Fig 4 and S3 Table).

**Fig 5:**
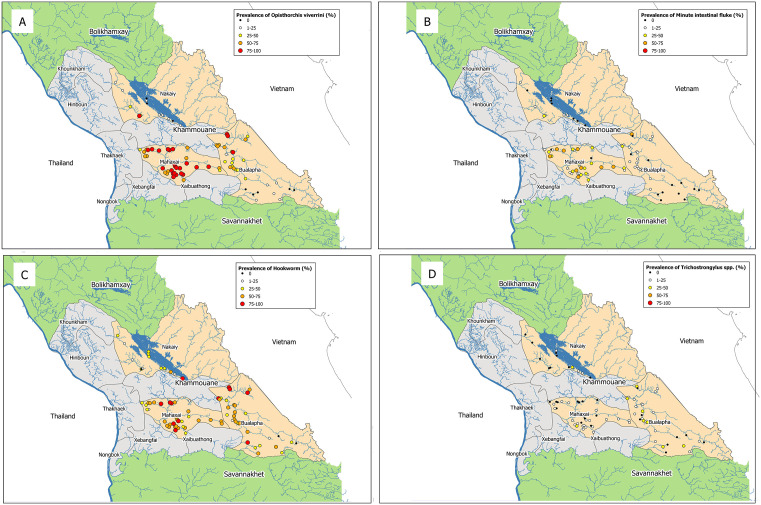
Prevalence of helminth infections per village: A) O.viverrini; B) Minute Intestinal Fluke; C) Hookworm; D) Trichostrongylus spp.; with prevalences of respectively 0% (black dot), 1-25% (white dot), 26-50% (yellow dot), 51-75% (orange dot) and 76-100% (red dot). The map was created using QGIS Geographic Information System (Open Source Geospatial Foundation Project. Version 3.4: https://qgis.org). The Lao administrative boundary shapefile was obtained from the geoBoundaries open license CC-BY 4.0 (https://www.geoboundaries.org/countryDownloads.html).

**Fig 6:**
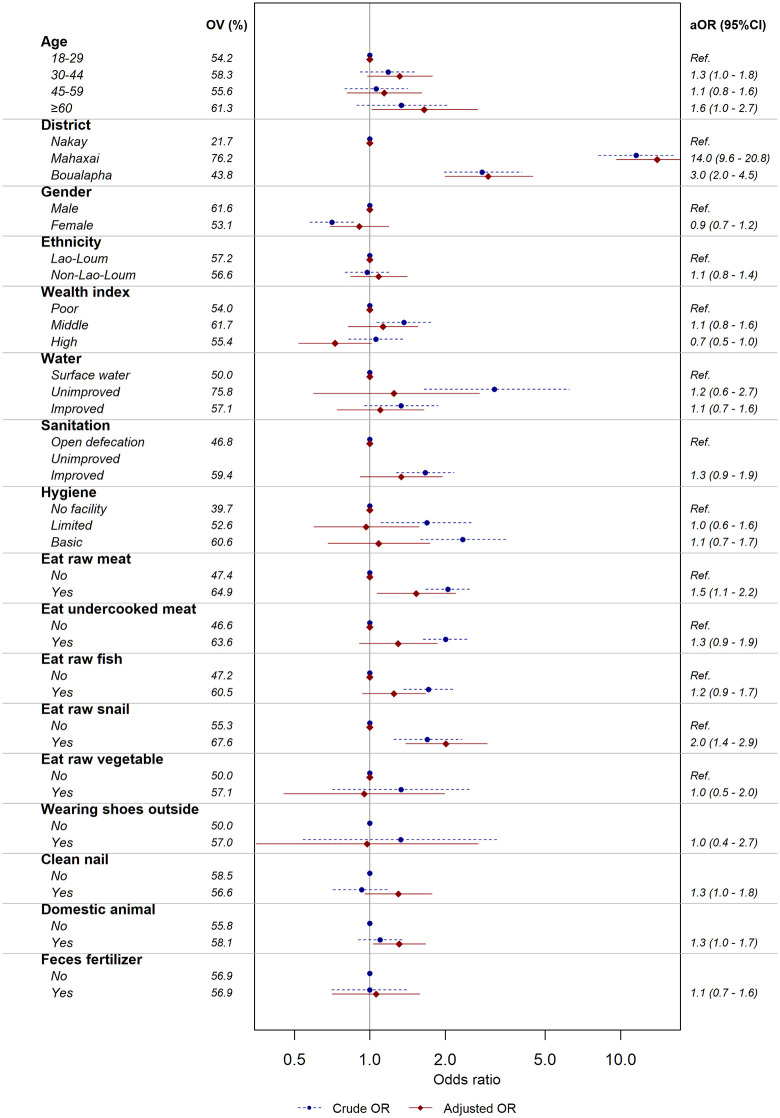
Factors associated with Opisthorchis viverrini (OV) infection among adults, n = 1530. Blue dotted line: bivariable analysis with crude odds ratio; Red solid line: multivariable analysis with adjusted odds ratio; 1.0 reference.

Sex-related differences were limited to specific parasites (MIF, Taenia spp.) rather than universal (Fig 8). In contrast, marked geographic heterogeneity was observed between districts. For example, Nakaiy district exhibited the lowest *O. viverrini* infection burden, with a prevalence of only about one-third that observed in Mahaxai district (Figs 5 and 6).

The pronounced geographic heterogeneity is further illustrated by the spatial distribution maps (Fig 5), which demonstrate a high infection burden in the southern districts of Mahaxai and Bualapha, whereas markedly lower prevalence rates are observed in Nakaiy.

### Distribution among district and special heterogeneity

The highest prevalence was typically found Mahaxai and Bualapha district, whereas a substantially lower prevalence was observed in Nakaiy district (Fig 5).

### Association of WASH levels and additional risk factors with intestinal helminth infections

**Fig 7:**
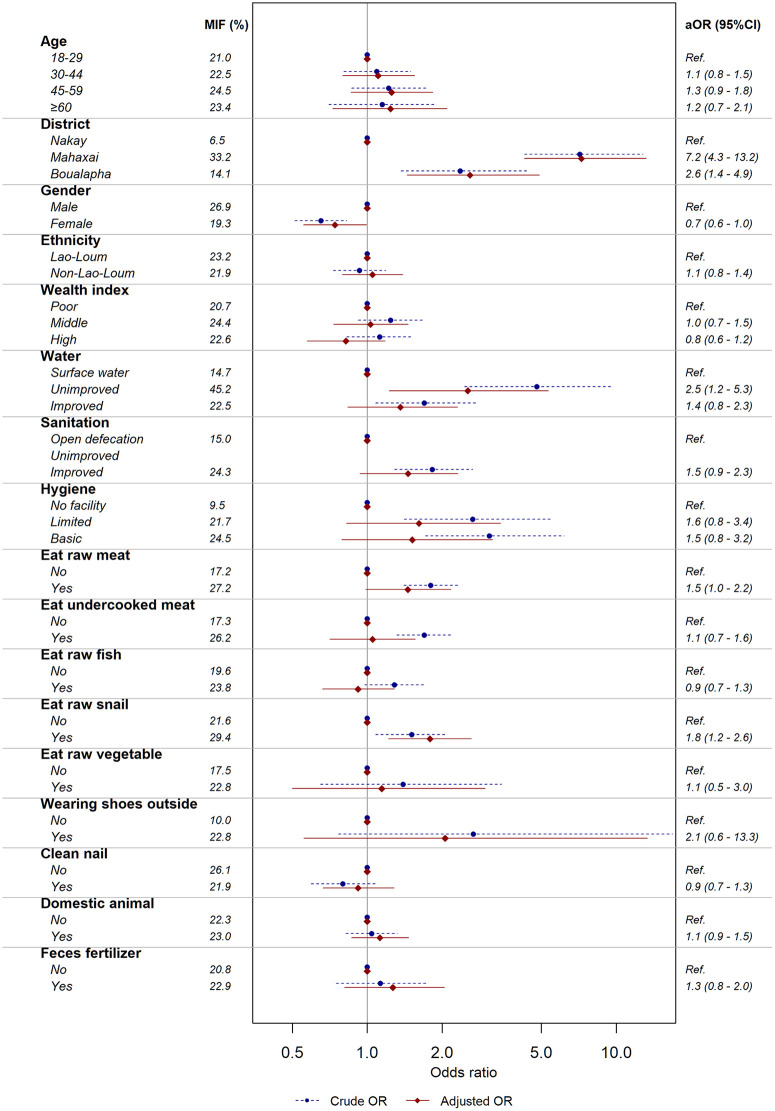
Factors associated with minute intestinal fluke (MIF) infection among adults, n = 1530. Blue dotted line: bivariable analysis with crude odds ratio; Red solid line: multivariable analysis with adjusted odds ratio; 1.0 reference.

**Fig 8:**
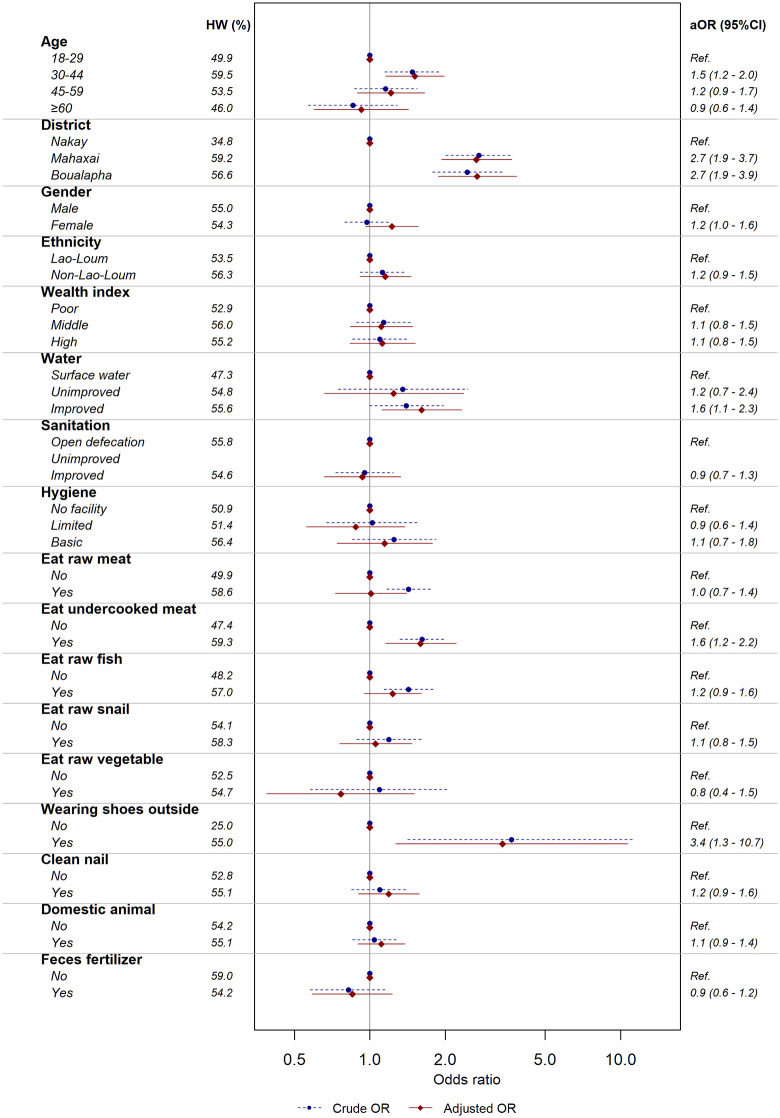
Factors associated with hookworm (HW) infection among adults, n = 1530. Blue dotted line: bivariable analysis with crude odds ratio; Red solid line: multivariable analysis with adjusted odds ratio; 1.0 reference.

**Fig 9:**
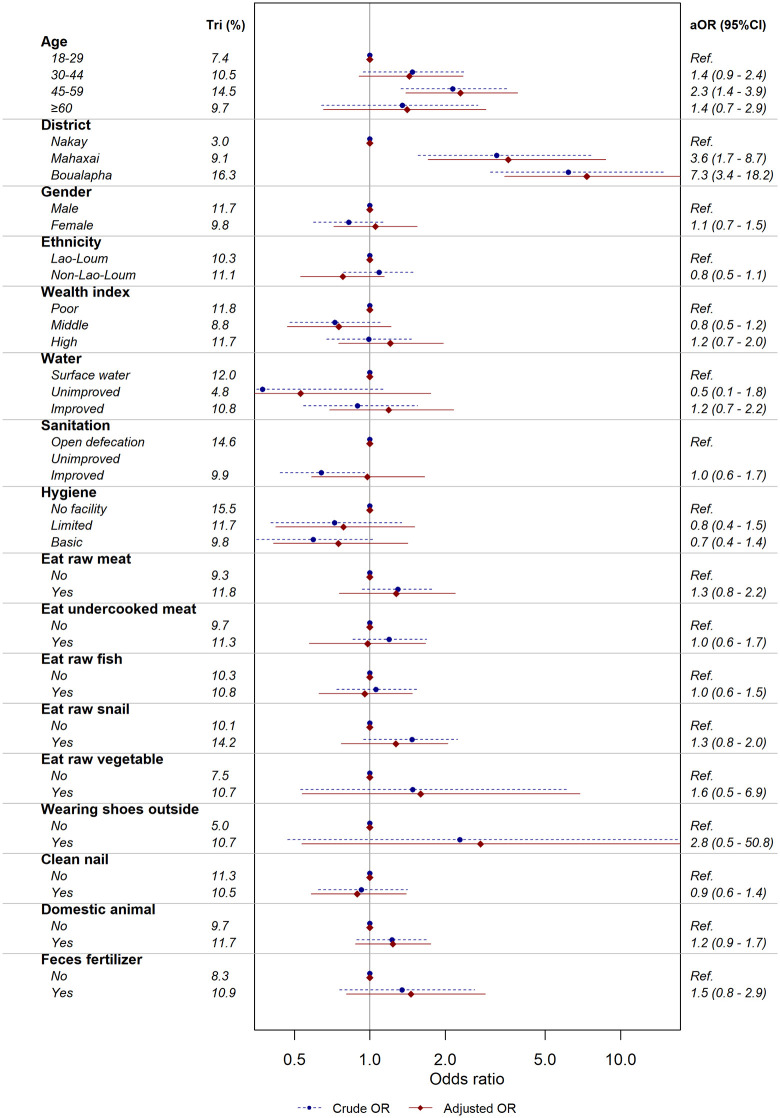
Factors associated with Trichostrongylus spp. (Tri) infection among adults, n = 1530. Blue dotted line: bivariable analysis with crude odds ratio; Red solid line: multivariable analysis with adjusted odds ratio; 1.0 reference.

Overall, no statistically significant associations were identified between WASH levels and any of the four main helminth infections (Figs 6−9). E.g., for *O. viverrini*, access to improved drinking water was not associated with infection risk (aOR = 1.1; 95% CI: 0.7–1.6; p = 0.634). Similarly, improved sanitation did not demonstrate a significant association with *O. viverrini* infection (aOR = 1.3; 95% CI: 0.7–1.6; p = 0.135). In addition, the presence of a basic sanitary facility at home was not related to *O. viverrini* infection (OR=1.1; 95%CI = 0.7–1.7; p = 0.736).

However, several other factors were statistically significantly associated with helminth infections. District of residence emerged as a significant determinant of infection. Residents of Mahaxai and Bualapha districts had significantly higher odds of infection with *O. viverrini*, MIF, hookworm, and *Trichostrongylus* spp. compared to those living in Nakaiy district. Mahaxai showed the highest odds ratios across all infections, particularly for *O. viverrini* (OR = 4.0; 95% CI: 1.9–8.6; p < 0.001) and MIF (OR = 7.2; 95% CI: 4.1–12.7; p < 0.001). Bualapha also demonstrated elevated risks, especially for *Trichostrongylus* spp*.* (OR = 7.3; 95% CI: 3.2–16.7; p < 0.001). Sex was also a significant predictor: women had a lower risk of MIF infection compared to men (OR = 0.7; 95% CI: 0.6–1.0; p = 0.045). Age stratification revealed that adults aged ≥60 years were significantly more likely to be infected with *O. viverrini* (OR = 1.6; 95% CI: 1.0–2.7; p = 0.044), while individuals aged 45–59 years had significantly higher odds of *Trichostrongylus* spp*.* infection (OR = 2.3; 95% CI: 1.4–3.8; p = 0.001) compared to younger age groups.

Among lifestyle-related factors, several behaviours were significantly associated with helminth infections. For *O. viverrini*, raw meat (OR = 1.5; 95% CI: 1.1–2.2; p = 0.021) and raw snail consumption (OR = 2.0; 95% CI: 1.4–2.9; p = 0.001), and feeding animals near the household (OR = 1.3; 95% CI: 1.0–1.7; p = 0.028) were identified as statistically significant risk factors. Raw snail consumption was also significantly associated with MIF infection (OR = 1.8; 95% CI: 1.2–2.6; p = 0.003). In addition, hookworm infection was significantly associated with the use of an improved water source (OR = 1.6; 95% CI: 1.1–2.3; p = 0.011), consumption of undercooked meat (OR = 1.6; 95% CI: 1.1–2.2; p = 0.005), and wearing shoes outdoors (OR = 3.4; 95% CI: 1.2–9.6; p = 0.020). We could not find any statistically significant association between *Trichostrongylus* spp*.* infection and any risk factor (Fig 9).

## Discussion

Our results revealed a very high burden of intestinal helminth infections – both trematodes and nematode infection – among participants. Specifically, *O. viverrini* and MIF infections were detected in 56.9% and 22.7% of individuals, respectively. Hookworm and *Trichostrongylus* spp*.* infections were found in 54.7% and 10.7% of the study population, respectively. Most of the infections were of light infection intensity.

The high prevalence of *O. viverrini* infection is a major public health concern. Chronic *O. viverrini* infection is associated with severe hepatobiliary morbidity, including the development of cholangiocarcinoma, a highly fatal bile duct cancer [21,22]. Indeed, one recent study indicated a high prevalence of suspected cholangiocarcinoma (7.2%) in rural Laos [22] *O. viverrini* endemicity has been reported across all provinces of the Lao PDR [11], but particularly elevated prevalence in regions where consumption of raw fish dishes and traditional fermented fish sauces is deeply embedded in local culture [23]. Furthermore, the 2019 national health survey, conducted across 17 provinces among adults aged 18 years and above, reported a prevalence of 18.8% [11]. Although the heavy burden of *O. viverrini* infection in Lao PDR is well recognized, the national helminth control program currently lacks the capacity to provide adequate public health interventions – such as access to praziquantel treatment – to all affected regions, except in two districts of Champasack Province where *O. viverrini* is co-endemic with *Schistosoma mekongi* [24].

Almost half (54.7%) of participants in our study were infected with hookworm. Although hookworm infection is most reported among children [25], its high prevalence among adults in these rice-farming communities is not unexpected, given their frequent and prolonged contact with soil. Similarly high prevalence rates have been documented in other studies in the Lao PDR; for instance, an 86.6% prevalence was reported among adults in Saravane Province [21]. The national helminth control program currently provides annual hookworm treatment; however, this intervention is limited to school-aged children [26].

The WASH levels observed among study households were moderate. Overall, 86.0% of participants had access to an improved water source, 82.3% utilized improved sanitation facilities, and 69.2% practiced basic hygiene. Conversely, only 9.9% relied on surface water, and 8.5% had no sanitation facility at home, but 17.7% practiced open defecation. Data from the Lao Social Indicator Survey (LSIS) 2023 [9] reported 8.4% of the population using surface water, 9.3% lacking sanitation facilities, and 12.8% practicing open defecation nationwide. Our study population’s WASH levels were similar to the reported levels in Khammouane province, which showed 12.9% of the population using surface water, 29.2% practicing open defecation and 23.1% having no facilities at home.

Several factors may have contributed to the lack of association in our study. First, we assessed WASH conditions only at participants’ households, assuming that their primary exposure occurred there. This approach did not account for participants’ mobility or potential WASH exposures in other settings. Second, the WASH intervention primarily focused on the provision and use of water and sanitation facilities at the household level, combined with health education messages. Diagnosis and treatment of helminth infections were not included; consequently, existing infections likely persisted even among participants benefiting from improved sanitation. Helminths can have a long lifespan – for example, *O. viverrini* may survive for up to 10 years [27]. Third, both the assessment of WASH levels (based on reported in interviews) and the detection of helminth infection (using the FECT method) rely on diagnostic tools with relatively low sensitivity. Therefore, misclassification of both exposure and outcome cannot be ruled out and may have contributed to the observed lack of association.These findings suggest that improvements in WASH infrastructure alone are insufficient to interrupt transmission. Additional, targeted intervention measures are required—such as reducing soil exposure to limit hookworm transmission and improving food hygiene practices to decrease food-borne trematode infections.However, we identified several other risk factors that were statistically significantly associated with helminth infections. Although intestinal helminth infections were endemic across all study districts, prevalence was particularly high in Mahaxai District. Mahaxai exhibited the highest infection rates, with 76.2% for *O. viverrini*, 29.5% for MIF, and 53.8% for hookworm. These results are consistent with earlier data from Mahaxai District (1996), which reported a helminth prevalence of 77.3% among children under 15 years, including 37.5% co-infected with hookworm and *O. viverrini* [28]. This indicates that intestinal helminth infections remain highly endemic in the area despite WASH interventions.

The diagnosis of *Trichostrongylus* spp. infection in our study population was unexpected. *Trichostrongylus* eggs were differentiated from hookworm eggs based on morphological characteristics, primarily egg size and shape, as observed by experienced laboratory technicians. To ensure quality, results were cross-checked and discussed among technicians; however, some misclassification cannot be ruled out due to the similarity between eggs. *Trichostrongylus* spp. are primarily nematodes of herbivorous animals such as sheep, goats, and cattle [29] and only occasionally infect humans [30]. Although a few human cases of *Trichostrongylus* infection have previously been reported in the Lao PDR [31], we detected this zoonotic nematode in more than 10% of our study participants; nearly 20% of those from Bualapha and 9% in Mahaxai districts. Our risk factor assessment did not identify any significant additional determinants of infection. Consequently, it is not possible to specify which transmission routes are predominant in these districts – whether through direct contact with herbivorous animals and/or through consumption of contaminated vegetables – both of which have been reported as potential routes of transmission in earlier studies [31]. However, we found that *Trichostrongylus* spp. is associated with older age groups, whereas a study in Salvador city, Brazil, reported the highest infection rate was observed among young age group 11–20 years [32].

In our study, we confirmed that age and sex are important determinants of helminth infection. Infection risk increased with age and was influenced by gender-specific behaviours. Individuals aged 60 years and older had a higher risk of *O. viverrini* infection, consistent with findings from previous studies, e.g., a study conducted in a rural community along the Mekong River on the Thai–Lao border in northeastern Thailand, which also reported an age-related increase in infection rates [33]. Our results further showed that women had a lower risk of MIF infection, consistent with previous studies in Thailand [34,35]. This reduced risk is likely attributable to behavioural differences, as women may be less likely to engage in high-risk dietary practices such as consuming raw or undercooked dishes, which are the primary sources of infection and which, in our study, were associated with increased helminth infection risk. Additionally, the cumulative nature of exposure over time explains the higher infection rates observed among older individuals.

In this study, we observed several counterintuitive associations between WASH indicators and intestinal helminth infections. Notably, the use of an improved water source was statistically significantly associated with a higher risk of hookworm infection. This finding is unexpected, as hookworm transmission is typically associated with inadequate sanitation and soil exposure rather than water source quality [36]. The unexpected association between improved water sources and higher hookworm infection may be explained by residual confounding. For instance, individuals with improved water access may still be predominantly engaged in agricultural activities, such as rice farming, which increases exposure to contaminated soil. Furthermore, access to improved water does not necessarily imply adequate sanitation or hygiene practices, both of which are critical in reducing soil-transmitted helminth infections.

Similarly, we unexpectedly found that wearing shoes was associated with an increased risk of hookworm infection. This result contradicts the widely accepted understanding that wearing footwear is a key preventive measure against hookworm transmission by reducing direct skin contact with contaminated soil [37]. Given that the vast majority of participants were farmers, we were unable to perform stratified analyses by occupation. Therefore, the observed association between footwear use and hookworm infection should be interpreted with caution, as it may reflect confounding within a relatively homogeneous occupational group.

Our study has several limitations. Firstly, late of information on the precise date of the WASH interventions in the study areas limits the interpretation of the results in relation to time since intervention. The geographical proximity of the three study districts—each with varying WASH levels but located within the same province—may have introduced selection bias by limiting the diversity of environmental and socio-economic conditions, thereby affecting the generalizability of our findings. Nonetheless, this setting enabled the analysis of a relatively homogeneous population under comparable environmental and WASH conditions. Furthermore, the random selection of villages likely ensured that the sample was representative of these three districts. Logistical constraints prevented the inclusion of 11 remote villages, particularly those in highland regions. Consequently, these socioeconomically disadvantaged areas were underrepresented, which may have led to an underestimation of the full range of socio-economic disparities and environmental exposures. Moreover, species-level differentiation of hookworm infections was not performed, as morphological methods cannot reliably distinguish *Ancylostoma* spp. from *Necator americanus*. In addition, differentiation between hookworm and *Trichostrongylus* eggs based on morphology may be imperfect. More accurate identification of both would require molecular techniques (e.g., PCR), which were not used in this study. Finally, reliance on self-reported WASH data may have introduced information bias, as participants could have provided ‌‌inaccurate or incomplete information about their practices and living conditions.

## Conclusion

Our study documents helminth infection levels of a significant public health concern in the study districts of Khammouane province, with a notably high prevalence of *O. viverrini* infections, along with hookworm and *Trichostrongylus* spp. Geographic location, age, sex, consumption of raw or undercooked food were linked to helminth infections. By contrast, improved WASH levels were not found to be associated with reduced helminth infections. To effectively reduce helminth infection, comprehensive control program is needed, including treatment and health education to promote safe food consumption in addition to ‌‌WASH improvements.

## Supporting information

S1 Data Data. (XLSX)

S1 Table Socio-demographics characteristics of study participants, n=1513. (DOCX)

S2 Table Reported risk factors of adult study participants (n=1530). (DOCX)

S3 Table Intestinal helminth infection prevalence and intestinal by district stratified by gender and age, n=1530. (DOCX)
