# Supplementary material for: High infection risk of intestinal helminths despite WASH interventions: A cross-sectional study in Khammouane province, Lao PDR
Source: PLoS Negl Trop Dis. 2026 Jun 1;20(6):e0014388. doi: 10.1371/journal.pntd.0014388 (PMC13245863; doi:10.1371/journal.pntd.0014388)
Supplement: S1 Table — (DOCX) [file pntd.0014388.s002.docx]

S1 Table: Socio-demographics characteristics of study participants, n=1513.

| **Variables** | | **Total** |
| --- | --- | --- |
|  | | N=1513 (%) |
| **Water assessment level** | |  |
| *Surface water* | | 150 (9.9) |
| *Unimproved* | | 62 (4.1) |
| *Improved* | |  |
|  | *Limited* | 364 (24.1) |
|  | *Basic* | 88 (5.8) |
|  | *Safely managed* | 849 (56.1) |
| **Sanitation assessment level** | |  |
| *Open defecation* | | 267 (17.7) |
| *Unimproved* | | 0 (0.0) |
| *Improved* | |  |
|  | *Limited* | 171 (11.3) |
|  | *Basic* | 403 (26.6) |
|  | *Safely managed* | 672 (44.4) |
| **Hygiene assessment level** | |  |
| *No facility* | | 116 (7.7) |
| *Limited* | | 350 (23.1) |
| *Basic* (Handwashing with soap) | | 1047 (69.2) |
