# Supplementary material for: High infection risk of intestinal helminths despite WASH interventions: A cross-sectional study in Khammouane province, Lao PDR
Source: PLoS Negl Trop Dis. 2026 Jun 1;20(6):e0014388. doi: 10.1371/journal.pntd.0014388 (PMC13245863; doi:10.1371/journal.pntd.0014388)
Supplement: S2 Table — (DOCX) [file pntd.0014388.s003.docx]

S2 Table: Reported risk factors of adult study participants (n=1530).

| **Variables** | **Total** |
| --- | --- |
|  | **N=1530 (%)** |
| ***Water source*** |  |
| Sufficient water |  |
| No | 407 (26.6) |
| Yes | 1123 (73.4) |
| Enough of drinking water |  |
| No | 340 (22.3) |
| Yes | 1190 (77.8) |
| Flooding at home |  |
| No | 1098 (71.8) |
| Yes | 432 (28.3) |
| Flooding source, (n=432) |  |
| River | 345 (79.9) |
| Lake | 70 (16.2) |
| Pond | 8 (1.9) |
| Other | 9 (2.1) |
| ***Food consumption risk*** |  |
| Eating raw meat |  |
| No | 696 (45.5) |
| Yes | 834 (54.5) |
| Freq. of eating raw meat |  |
| <2tpw | 615 (73.7) |
| 1-5tpm | 190 (22.8) |
| >5tpm | 29 (3.5) |
| Eating uncooked or undercooked meat |  |
| No | 601 (39.3) |
| Yes | 929 (60.7) |
| Freq. of eating undercooked meat |  |
| <2tpw | 624 (67.2) |
| 1-5tpm | 257 (27.7) |
| >5tpm | 48 (5.2) |
| Eating raw fish |  |
| No | 409 (26.7) |
| Yes | 1121 (73.3) |
| Freq. of eating raw fish |  |
| <2tpw | 371 (33.1) |
| 1-5tpm | 332 (29.6) |
| >5tpm | 418 (37.3) |
| Eating raw shellfish |  |
| No | 1444 (94.4) |
| Yes | 86 (5.6) |
| Freq. of eating raw shellfish |  |
| <2tpw | 64 (74.4) |
| 1-5tpm | 18 (20.9) |
| >5tpm | 4 (4.7) |
| Eating raw snail |  |
| No | 1326 (86.7) |
| Yes | 204 (13.3) |
| Freq. of eating raw snail |  |
| <2tpw | 163 (79.9) |
| 1-5tpm | 27 (13.2) |
| >5tpm | 14 (6.9) |
| Eating raw veggies |  |
| No | 40 (2.6) |
| Yes | 1490 (97.4) |
| Freq. of eating raw veggies |  |
| <2tpw | 100 (6.7) |
| 1-5tpm | 295 (19.8) |
| >5tpm | 1095 (73.5) |
| Eating remaining foods |  |
| No | 355 (23.2) |
| Yes | 1175 (76.8) |
| Keeping unfinished foods, (n=1175) |  |
| Inside the fridge | 871 (74.1) |
| Outside the fridge | 69 (5.9) |
| Leave it with cover | 229 (19.5) |
| Other | 6 (0.5) |
| ***Environmental risk*** |  |
| Wearing shoes outside |  |
| No | 20 (1.3) |
| Yes | 1510 (98.7) |
| Cleaning nails |  |
| No | 284 (18.6) |
| Yes | 1246 (81.4) |
| ***Contact with animal*** |  |
| Animal near house |  |
| No | 792 (51.8) |
| Yes | 738 (48.2) |
| Feeding pig |  |
| No | 807 (52.8) |
| Yes | 723 (47.3) |
| Slaughtering pig |  |
| No | 1179 (77.1) |
| Yes | 351 (22.9) |
| Faeces as fertilizers |  |
| No | 144 (9.4) |
| Yes | 1386 (90.6) |
| ***History of illness*** |  |
| Taken anti-parasites |  |
| No | 1080 (70.6) |
| Yes | 450 (29.4) |
| Being ill in the last 6 months |  |
| No | 1011 (66.1) |
| Yes | 519 (33.9) |
| If yes, symptoms, n=519 |  |
| Symptoms not related to gastrointestinal diseases | 282 (54.3) |
| Symptoms related to gastrointestinal diseases | 237 (45.7) |

**tpw = times per week**

**tpm = times per month**
