# Supplementary material for: High infection risk of intestinal helminths despite WASH interventions: A cross-sectional study in Khammouane province, Lao PDR
Source: PLoS Negl Trop Dis. 2026 Jun 1;20(6):e0014388. doi: 10.1371/journal.pntd.0014388 (PMC13245863; doi:10.1371/journal.pntd.0014388)
Supplement: S3 Table — (DOCX) [file pntd.0014388.s004.docx]

S3 Table: Intestinal helminth infection prevalence and intestinal by district stratified by gender and age, n=1530.

| **Parasites** | **n (%)** | **District** | | | **Gender** | | **Age groups (Years)** | | | | |
| --- | --- | --- | --- | --- | --- | --- | --- | --- | --- | --- | --- |
|  |  | Nakaiy | Mahaxai | Bualapha | Male | Female | 18 to 20 | 21 to 30 | 31 to 40 | 41 to 50 | >50 |
|  | (n=1530) | (n=230) | (n=777) | (n=523) | (n=685) | (n=845) | (n=65) | (n=336) | (n=481) | (n=339) | (n=309) |
| **Trematodes** |  |  |  |  |  |  |  |  |  |  |  |
| *Opisthorchis viverrini* | 871 (56.9) | 50 (21.7) | 592 (76.2) | 229 (43.8)** | 422 (61.6) | 449 (53.1)** | 33 (50.8) | 186 (55.4) | 275 (57.2) | 196 (57.8) | 181 (58.6) |
| *Minute intestinal fluke* | 347 (22.7) | 15 (6.5) | 258 (33.2) | 74 (14.2)** | 184 (26.9) | 163 (19.3)** | 13 (20.0) | 70 (20.1) | 113 (23.5) | 73 (21.5) | 78 (25.2) |
| *Paragonimus* spp. | 2 (0.1) | 0 (0.0) | 1 (0.1) | 1 (0.2) | 2 (0.3) | 0 (0.0) | 0 (0.0) | 1 (0.3) | 0 (0.0) | 0 (0.0) | 1 (0.3) |
| *Large trematode eggs^#^* | 21 (1.4) | 2 (0.9) | 2 (0.3) | 17 (3.3) | 14 (2.0) | 7 (0.8) | 2 (3.1) | 3 (0.9) | 6 (1.2) | 6 (1.8) | 4 (1.3) |
| **Nematodes** |  |  |  |  |  |  |  |  |  |  |  |
| Hookworm | 836 (54.7) | 80 (34.8) | 460 (59.2) | 296 (56.6)** | 377 (55.0) | 459 (54.3) | 31 (47.7) | 169 (50.3) | 282 (58.6) | 192 (56.6) | 162 (52.4) |
| *Trichostrongylus* spp. | 163 (10.7) | 7 (3.0) | 71 (9.1) | 85 (16.3)** | 80 (11.7) | 83 (9.8) | 4 (6.2) | 25 (7.4) | 54 (11.2) | 45 (13.3) | 35 (11.3) |
| *Trichuris trichiura* | 67 (4.4) | 1 (0.4) | 10 (1.3) | 56 (10.7)** | 41 (6.0) | 26 (3.1) | 1 (1.5) | 15 (4.5) | 25 (5.2) | 16 (4.7) | 10 (3.2) |
| *Strongyloides stercoralis* | 73 (4.8) | 10 (4.4) | 35 (4.5) | 28 (5.4) | 45 (6.6) | 28 (3.3)* | 0 (0.0) | 12 (3.6) | 26 (5.4) | 19 (5.6) | 16 (5.2) |
| *Ascaris lumbricoides* | 18 (1.2) | 3 (1.3) | 2 (0.3) | 13 (2.5)** | 6 (0.9) | 12 (1.4) | 1 (1.5) | 3 (0.9) | 9 (1.9) | 3 (0.9) | 2 (0.7) |
| *Enterobius vermicularis* | 12 (0.8) | 1 (0.4) | 9 (1.2) | 2 (0.4) | 6 (0.9) | 6 (0.7) | 0 (0.0) | 1 (0.3) | 3 (0.6) | 6 (1.8) | 2 (0.7) |
| *Physaloptera* spp. | 6 (1.2) | 0(0.0) | 3 (0.4) | 3 (0.6) | 3 (0.4) | 3 (0.4) | 0 (0.0) | 1 (0.3) | 2 (0.4) | 1 (0.3) | 2 (0.7) |
| **Cestodes** |  |  |  |  |  |  |  |  |  |  |  |
| *Taenia* spp. | 119 (7.8) | 5 (2.2) | 71 (9.1) | 43 (8.2)** | 76 (11.1) | 43 (5.1)** | 1 (1.5) | 23 (6.9) | 39 (8.1) | 25 (7.4) | 31 (10.0) |
| *Hymenolepis diminuta* | 12 (0.8) | 2 (0.9) | 8 (1.0) | 2 (0.4) | 6 (0.9) | 6 (0.7) | 2 (3.1) | 2 (0.6) | 1 (0.2) | 3 (0.8) | 4 (1.3) |
| *Diphyllobothrium latum* | 1 (0.07) | 0 (0.0) | 0 (0.0) | 1 (0.2) | 0 (0.0) | 1 (0.1) | 0 (0.0) | 1 (0.3) | 0 (0.0) | 0 (0.0) | 0 (0.0) |
| **Mono/polyparasitism** |  |  |  |  |  |  |  |  |  |  |  |
| No infection | 301 (19.7) | 106 (46.1) | 85 (10.9) | 110 (21.0) | 105 (15.3) | 196 (23.2) | 16 (24.6) | 82 (24.4) | 92 (19.1) | 58 (17.1) | 53 (17.2) |
| Single infection | 411 (26.9) | 83 (36.1) | 179 (23.0) | 149 (28.5) | 172 (25.1) | 239 (28.3) | 23 (35.4) | 96 (28.6) | 113 (23.5) | 93 (27.4) | 86 (27.8) |
| Double infection | 461 (30.1) | 33 (14.4) | 279 (35.9) | 149 (28.5) | 212 (31.0) | 249 (29.5) | 18 (27.7) | 83 (24.7) | 152 (31.6) | 112 (33.0) | 96 (31.1) |
| Triple infection | 256 (16.7) | 7 (3.0) | 168 (21.6) | 81 (15.5) | 141 (20.6) | 115 (13.6) | 6 (9.2) | 55 (16.4) | 87 (18.1) | 51 (15.0) | 57 (18.5) |
| Quadruple infection | 101 (6.6) | 1 (0.4) | 66 (8.5) | 34 (6.5) | 55 (8.0) | 46 (5.4) | 2 (3.0) | 20 (6.0) | 37 (7.7) | 25 (7.4) | 17 (5.5) |
| *<0.05, ** <0.001, P-value were using the Chi-square test | | | | | |  |  |  |  |  |  |
